# A Truncated Multi-Thiol Aptamer-Based SARS-CoV-2 Electrochemical Biosensor: Towards Variant-Specific Point-of-Care Detection with Optimized Fabrication

**DOI:** 10.3390/bios15010024

**Published:** 2025-01-06

**Authors:** Sergio Roberto Molina Ramirez, Nafiseh Samiseresht, Mateo Alejandro Martínez-Roque, Ferdinando Catania, Kevin Graef, Martin Rabe, Andreas Offenhäusser, Dirk Mayer, Gabriela Figueroa-Miranda

**Affiliations:** 1 Institute of Biological Information Processing, Bioelectronics (IBI-3), Forschungszentrum Jülich GmbH, 52428 Jülich, Germany; sergio.molina-ramirez@ukbonn.de (S.R.M.R.); mam4088@med.cornell.edu (M.A.M.-R.); fer.catania@studenti.unina.it (F.C.); k.graef@fz-juelich.de (K.G.); a.offenhaeusser@fz-juelich.de (A.O.); dirk.mayer@fz-juelich.de (D.M.); 2 Department of Interface Chemistry and Surface Engineering, Max Planck Institute for Sustainable Materials GmbH, 40237 Düsseldorf, Germany; n.samiseresht@mpie.de (N.S.); m.rabe@mpie.de (M.R.)

**Keywords:** electrochemical aptasensor, SARS-CoV-2, aptamer truncation, multi-thiol, S protein

## Abstract

With the goal of fast and accurate diagnosis of infectious diseases, this study presents a novel electrochemical biosensor that employs a refined aptamer (C9t) for the detection of spike (S) protein SARS-CoV-2 variants in a flexible multielectrode aptasensor array with PoC capabilities. Two aptamer modifications were employed: removing the primer binding sites and including two dithiol phosphoramidite anchor molecules. Thus, reducing fabrication time from 24 to 3 h and increasing the stability and sparseness for multi-thiol aptasensors compared to a standard aptasensor using single thiols, without a reduction in aptamer density. The biosensor fabrication, optimization, and detection were verified in detail by electrochemistry, QCM-D, SPR, and XPS. The analyte–receptor binding was further confirmed spectroscopically at the level of individual molecules by AFM-IR. The aptasensor possesses a low limit of detection (8.0 fg/mL), the highest sensitivity reported for S protein (209.5 signal per concentration decade), and a wide dynamic detection range (8.0 fg/mL–38 ng/mL) in nasopharyngeal samples, covering the clinically relevant range. Furthermore, the C9t aptasensor showed high selectivity for SARS-CoV-2 S proteins over biomarkers for MERS-CoV, RSV, and Influenza. Even more, it showed a three times higher sensitivity for the Omicron in comparison to the Wuhan strain (wild type), alpha, and beta variants of the SARS-CoV-2 virus. Those results demonstrate the creation of an affordable and variant-selective refined C9t aptasensor that outperformed current rapid diagnosis tests.

## 1. Introduction

In December 2019, the world was shaken by the appearance of a novel strain of coronaviruses, SARS-CoV-2, the severe acute respiratory syndrome coronavirus 2. Through its rapid spread, more than 777 million infections and 7 million related deaths have been recorded by the World Health Organization (WHO) as a result of the coronavirus disease 2019 (COVID-19) until November 2024.

The spread caused a global pandemic leading to devastating consequences in virtually every aspect of regular life. Three factors contributed drastically to this spread: such a virus has never infected humans in such an uninterrupted form, its high transmission rate, and its relatively high morbidity and mortality [1,2]. In the past decade, there have been endemic outbreaks as well of other highly contagious or deadly diseases such as Influenza A H1N1 “swine” influenza (2009), chikungunya (2014), Zika (2015), and Ebola (2014 to present). The COVID-19 pandemic was caused by a coronavirus, from which multiple forms have been already circulating locally in different regions of the world [3]. In the last two decades, major outbreaks of specific strains of these viruses have been observed in the form of the severe acute respiratory syndrome coronavirus (SARS-CoV) in 2002–2003 and the Middle East respiratory syndrome coronavirus (MERS-CoV), followed by the recent pandemic through SARS-CoV-2. The causes of these dangerous situations are “multifaceted, complex, and deserving of serious examination” [2].

Therefore, pandemic prevention and treatment require multifaceted solutions, one of its pinnacles being the timely recognition of the pathogen and those infected. Once identified, governments, health organizations, and physicians should have the ability to break transmission lines before they can become pandemic [4]. The viral testing required should be fast, low-cost, and mobile to facilitate point-of-care (PoC) measurement without requiring external equipment and trained personnel [5]. However, after the 2019 pandemic, many governments are no better prepared for a new pandemic than before. For instance, no uniform quantitative rapid diagnostic test platforms are available, and further research and development are required in this area.

For SARS-CoV-2, multiple tests have been developed. The gold standard is based on the amplification of genetic material with real-time polymerase chain reaction (RT-PCR) with high specificity and sensitivity [6]. This method, however, requires a multi-step process, which can only be conducted in specialized laboratories with the necessary equipment and trained personnel. Furthermore, the testing period lasts around 3–4 h including sample preparation and gene amplification. These conditions limit the use of this method as a PoC test [7]. Lateral flow assays (LFA) are the second most used approach for detecting SARS-CoV-2 infection, they are fast and affordable. However, they have limited sensitivity and selectivity and provide no quantitative information so additional test methods are still needed, to confirm the strain and the viral load [8,9,10].

To address PoC testing the WHO introduced the ASSURED Criteria: Affordable, Sensitive, Specific, User-friendly, Rapid and robust, Equipment-free, and Deliverable to end users [11]. Different approaches that could satisfy some of the WHO requirements have been developed in the form of immunoassays [12], field-effect transistor (FET) biosensors [13], opto-microfluidic sensors [14], surface-enhanced Raman scattering (SERS) sensors [15], and electrochemical sensors [7,16,17,18,19]. All have delivered promising results; however, each approach has its specific limitations, such as relatively high cost, thermal instability as in immunoassays, or a requirement for laboratory equipment as in opto-microfluidic or SERS sensors. Out of these methods, electrochemical sensors meet the requirements for low-cost, mobile, and relatively ease-of-use testing methods [20].

For electrochemical sensors, a sensor receptor molecule and a signal transducer are needed, to transduce the presence of chemical analytes into a measurable electrical signal. State-of-the-art antibodies are some of the most commonly used biosensor receptor molecules. However, antibodies come with a set of hindrances. They are not thermally stable, have a high molecular weight, and present batch-to-batch variation and it is difficult, therefore, to conduct homogeneous essays. Furthermore, their production generally involves animal suffering [21,22]. An alternative promising approach to pathogen testing and as an alternative to antibody-based assays is the use of aptamers as receptor molecules, which are short oligonucleotide or oligopeptide strands that have been designed to specifically bind their target molecule by the so-called SELEX process [23]. Aptamers promise to deliver a solution to the limitations of antibodies. They are small, with a high affinity, thermally stable, with high reproducibility, and lower fabrication costs. Furthermore, as synthetic molecules, they require just a general biochemistry lab to be produced, and their in vitro synthesis allows easier chemical modifications. Lastly, they are relatively simple to implement as receptors in several transducer platforms, for instance, in electrochemical biosensors [24,25]. This aptamer-based biosensor (aptasensor) approach promises to be highly sensitive, and selective and represents an even more stable option than current state-of-the-art tests [26]. These advantageous characteristics combined lead to the possibility of using the electrochemical aptasensor as a PoC device [20,22].

Electrochemical aptasensors have already been developed to detect pathogens, such as malaria [27] and SARS-CoV-2 [16,28,29]. This research aims to develop a low-cost, polymer-based multichannel aptasensor with optimized fabrication processes for the variant-specific detection of the Spike-Glycoprotein (S protein) of the SARS-CoV-2 virus. To this end, our novel aptamer underwent two critical modifications that could be seminal also for other aptasensor developments [25]. Firstly, its primer sequences, which are necessary for amplification during the SELEX process, were removed, leaving only the central random region, building on previous works [30,31,32,33]. Secondly, two dithiol phosphoramidite (di-DTPA) groups with four available anchoring thiol groups were introduced [34].

The economic advantage of aptamers is amplified when shorter sequences are used, reducing production costs. Importantly, shorter aptamers also enable binding events to occur closer to the electrode interface, allowing stronger detection signals. Additionally, long single-stranded DNA (ssDNA) exhibits film growth suboptimal for the detection of binding events [35]. We chose to remove the primer binding sites based on the existing literature, which suggests that aptamer binding is most likely determined by the aptamer’s random region [36,37,38]. Furthermore, the addition of di-DTPA groups promises to increase the stability of the receptor layer and avoids unspecific weak nitrogen–gold interactions, increasing thus the sparsity of the aptamer film [39].

Surface plasmon resonance (SPR), X-ray photoelectron spectroscopy (XPS), and atomic force microscope–infrared spectroscopy (AFM-IR) were used to confirm the functionalization of the truncated aptasensor and to determine the dissociation constant (*K*_D_) with the targets (Wuhan strain (wild-type) and Omicron strain S proteins). The fabrication time was optimized using a quartz crystal microbalance (QCM). Lastly, flexible multi-electrode arrays (flexMEAs) were used for the aptasensor fabrication and characterization, immobilizing aptamers on multiple electrodes to increase the reliability of the signal through the redundancy of each electrode’s individual signal. flexMEAs chips have been reported to have a low fabrication cost [27]. To corroborate the functionality of the aptasensor, its performance was characterized in different matrices, and its sensitivity and selectivity were evaluated with different variants of the SARS-CoV-2 S protein and proteins of other respiratory diseases. This thoroughly characterized and optimized aptasensor, demonstrated its capability as an affordable and reliable testing tool to manage future fast-spreading infections.

## 2. Materials and Methods

### 2.1. Materials and Instruments

Differential pulse voltammetry (DPV), cyclic voltammetry (CV), chronocoulometric (CC) measurements, and electrochemical cleaning were conducted on a CHI1030B potentiostat (CH Instruments, Austin, TX, USA). For all electrochemical experiments, a 3-electrode system was used. As reference electrode (RE) and counter electrode (CE), a saturated Ag/AgCl electrode (DriRef2, World Precision Instruments, Friedberg, Germany) and a platinum wire were used, respectively. All potentials reported here are referenced to Ag/AgCl. flexMEA chips with 16 individually addressable electrodes were used as working electrodes (WE). Those chips were fabricated by a low-cost fabrication process as recently reported by Figueroa-Miranda et al. [27] and briefly described in the Supplementary Material (Figure S1).

### 2.2. Chemicals and Solutions

Chemicals used for the solutions were procured from Sigma Aldrich (Darmstadt, Germany) and prepared in ultra-pure deionized water (18.2 MΩ cm, Milli-Q, Millipore, Merck, Darmstadt, Germany). The used solutions are as follows: 25 mM tris-HCl buffer (tris 25.0 mM with HCl 30% 25 mM and NaCl 0.1 M, pH 7.5), 10 mM tris-HCl (tris 10 mM with HCL 30% 10 mM and NaCl 0.1 M, pH 7.4), high salt 10 mM phosphate-buffered saline with magnesium chloride (PBS + Mg^2+^, 10.0 mM NaH_2_PO_4_/Na_2_HPO_4_ with 1 M NaCl and 1 mM MgCl_2_, pH 7.0–7.2), and 5 mM ferri/ferrocyanide (ferri/ferrocyanide solution, 25 mM tris-HCl, 5 mM potassium hexacyanoferrate trihydrate and 5 mM potassium hexacyanoferrate).

### 2.3. Analytes

The main target analyte was the wild-type S protein (S1+S2 ECD, His Tag) of the SARS-CoV-2 Virus (2019-nCoV, Sino Biological Europe GmbH, Eschborn, Germany). For selectivity experiments other respiratory viruses were used: the glycoprotein G of the respiratory syncytial virus (RSV-G Protein) [A, rsb1734], the hemagglutinin/HA1 protein of the Influenza A H1N1 virus (A/Mexico/InDRE4114/2009) and the S protein (S1+S2 ECD, as 1-1297) of the middle eastern respiratory syndrome coronavirus (MERS-CoV). All proteins were procured from Sino Biological Europe GmbH. The selectivity against different lineages of SARS-CoV-2 was also investigated by using the S protein of Beta (B.1.351), Alpha (B.1.1.7), and Omicron variants (all from antibodies-online GmbH, Aachen, Germany).

### 2.4. Functionalization Molecules

The aptamer C9 was developed through the SELEX method [25]. This aptamer was truncated by excluding the primer sequences; therefore, the aptamer here used is named C9t. This aptamer was functionalized on the 5′-end with 2 groups of dithiol phosphoramidite (di-DTPA, FRIZ Biochem, Neuried, Germany). The blocking molecule poly(ethylene glycol) methyl ether thiol 2000 (PEG 2000, Sigma-Aldrich, Darmstadt, Germany) was used in a 5 mg/mL concentration dissolved in tris-HCl 25 mM buffer. For the density measurements using chronocoulometry, the blocking molecule 6-mercapto-1-hexanol (MCH, Sigma-Aldrich, Darmstadt, Germany) was used instead of PEG as the latter interferes with those measurements. The sequence of the truncated C9t aptamer is as follows:

5′-DTPA_2_–GGG GGC GTC AAG CGG GGT CAC ATC GGA GTA GGG AAT CTT G-3′

### 2.5. Electrode Cleaning Procedure

The flexMEAs were extensively cleaned through a protocol reported by Zhang et al. [40] Briefly, using cyclic voltammetry (CV), the singular electrodes were cleaned using two different sweep voltages: a CV in 0.1 M NaOH, from −1.35 V to −0.35 V at a scan rate of 2 V/s with a total of 10 scans, followed by CV in 0.05 M H_2_SO_4_ from 0 V to 1.5 V at a scan rate of 1 V/s with 20 scans. Furthermore, for the electrochemical area calculation, 2 scans were recorded from 0 V to 1.5 V at a scan rate of 0.1 V/s in 0.05 M H_2_SO_4_ [41].

### 2.6. Electrode Functionalization

The disulfide groups of the DTPA were reduced through exposure to tris(2-carboxyethyl) phosphine 10 mM (TCEP, Sigma-Aldrich, Darmstadt, Germany) for 1 h at room temperature in a 1:3 volume ratio between the aptamer and TCEP solution. After reduction, the TCEP/aptamer solution was diluted into high salt 10 mM PBS + Mg^2+^ solution (see above) to a final volume of 2 mL. If not otherwise stated, aptamers were used in a 0.5 µM concentration. Consequently, the flexMEA, attached to a ZIF connector, was dipped into the aptamer solution and left for incubation for 2 h at room temperature. Following this, the flexMEA was rinsed with tris-HCL 25 mM and subsequently dipped into the PEG 2000/tris-HCL solution for 1 h. Lastly, the flexMEA was cleaned gently with a tris-HCl solution. Hereafter, the flexMEA was functionalized and ready for usage after a total preparation time of less than three hours.

### 2.7. Quartz Crystal Microbalance with Dissipation (QCM-D)

All QCM-D measurements were performed utilizing a QSense Explorer (Biolin Scientific, Västra Frölunda, Sweden) with the standard flow module. The flow into the chamber was kept constant for all experiments at 40 µL/min using a peristaltic pump (Model ISM596D, ISMATEC). First, the chambers were flushed with Milli-Q for 10 min. This initial cleaning was followed by an inflow of the blank solution (tris-HCl 25 mM). This flow was kept until the frequency shift was below ±0.2 Hz in a time window of 10 min. After stabilization, the measurement was started and the blank flowed for 15 min more, to have a stable 0 Hz baseline. Afterward, the change to the reagent solution (aptamer or blocking molecules) was carried out, and for the first 10 min, no recirculation was performed. After 10 min, a recirculation of the same solution was performed. Between the change of solutions, a cleaning step with the blank solution was conducted, until the signal change stayed at a maximum variance of ±0.5 Hz for 10 min. For calculating the mass change Δm, the measured frequency change Δf was used according to the Sauerbey equation,

(1) $$\Delta m=\frac{A\sqrt{\rho_{q}\cdot \mu_{q}}}{2f_{0}}\Delta f,$$

to calculate changes down to $\Delta m=10^{-10}\;g$; where $f_{0}$ is the resonance frequency, $A=0.7^{2}\cdot \pi \;{\mathrm{cm}}^{2}$ is the area of the used electrode, $\rho_{q}=2.648\;{g/\mathrm{cm}}^{3}$ is the density of the quartz crystal, and $\mu_{q}=2.947\times 10^{11}\;g/(s^{2}\cdot \mathrm{cm})$ is the shear modulus of the quartz for AT-cut crystal [42].

### 2.8. Surface Plasmon Resonance (SPR)

A BIAcore T200 instrument (GE Healthcare Europe, Düsseldorf, Germany) was employed for a single-cycle kinetic assay. His-tagged wild-type and Omicron variant S proteins were immobilized onto a Series S Sensor Chip NTA (GE Healthcare Europe, Düsseldorf, Germany) at a concentration of 148.8 nM (8 ng/μL). This immobilization was conducted at a flow rate of 10 μL/min at 25 °C using TNa7 buffer until the response units reached approx. 1200. Lines two and three of the sensor chip were designated for immobilizing the wild-type and Omicron variant proteins, respectively. Line one served as a reference with no protein immobilized. A concentration series for the aptamer C9t was prepared with the following points: 55, 166, 500, 1500, and 3000 nM. The association and dissociation times were set to 180 s and 600 s, respectively. Data was analyzed using BIAcore T200 evaluation software (version 3.2, GE Healthcare Europe, Düsseldorf, Germany) and fitted to a 1:1 binding stoichiometry model to determine the dissociation constant (*K*_D_).

### 2.9. Atomic Force Microscope–Infrared Spectroscopy (AFM-IR)

The activation and cleaning of the used Au (111) single crystal were carried out by thoroughly rinsing it in ethanol, isopropanol, and Milli-Q water. After drying, the crystal was annealed for 10 min in a hydrogen flame (orange glowing) and cooled down to room temperature in a nitrogen stream. The subsequent aptamer modification and PEG blocking were carried out as described in the previous section. Employing a Bruker NanoIR3 (Bruker Corporation, Billerica, MA, USA) in tapping mode, AFM-IR, imaging, and nano-IR point spectra were obtained. A tunable quantum cascade laser system (QCL, DRS Daylight Solutions, San Diego, CA, USA) with four modules covering the range 910–1800 cm^−1^ was used as an IR source. The lasers were operated at 10% of power (<0.1 W) in pulsed mode with 100 ns pulse width, and a pulse repetition rate of around 375 kHz for all measurements. Gold-coated silicon probes (nominal frequency 75 ± 15 kHz, Anasys Instruments, type: Tapping Mode NIR2 Probes for nanoIR2) were used. For studying the binding of the analyte to the receptor, 30 ng/mL of S protein was incubated on the receptor layer for 45 min, rinsed with buffer, dried, and measured by AFM-IR in a nitrogen environment. All spectra were recorded using an off-resonance detection described by [43]. The spectra are normalized at 954 cm^−1^ and averaged. In this paper, the range from 1430 to 1800 cm^−1^ (two modules) is shown covering the amide I and amide II bands of the attached protein.

### 2.10. Chronocoulometry (CC)

The voltage sweep was performed in buffer solution and buffer containing 10 mM [Ru(NH^3^)_6_]^3+^ solution from 0.2 V to −0.5 V with 2 steps, and a 2 ms sampling interval. This method was used to calculate the density of aptamers on the surface using the following

(2) $$\Gamma_{DNA}=\Gamma_{0}\cdot (z/m)\cdot N_{A},$$

where $\Gamma_{0}$ represents the amount of redox marker confined near the electrode surface. $\Gamma_{DNA}$ is the probe surface density in molecules/cm^2^, m is the number of bases in the probe DNA, z is the charge of the redox molecule, and $N_{A}$ is Avogadro’s number [44]. This procedure was conducted with MCH as a blocking molecule instead of PEG as foreseen by the original protocol in [44].

### 2.11. Differential Pulse Voltammetry (DPV)

In 5 mM ferri/ferrocyanide or buffer solution, scans were conducted from 0.0 V to 0.7 V with increments of 5 mV, an amplitude of 25 mV, a pulse width of 50 ms, a sampling width of 25 ms and a pulse period of 100 ms. The blank response DPV current signals of the sensor electrodes were measured without analyte exposure. Subsequently, the sensor responses were recorded after 30 min incubation for increasing target protein concentrations. The analyte response characteristic was then calculated in reference to the maximum blank response $I_{0}$, with

(3) $$\Delta I⁄I_{0}=(I_{i}-I_{0})/\;I_{0}\;,$$

where $I_{i}$ is the maximum current response after the analyte exposure window. For the analysis of the data obtained MATLAB (v. 9.13.0 (R2022b), The MathWorks Inc.: Natick, MA, USA) was used.

## 3. Results

### 3.1. Characterization of the Truncated Aptasensor

A previously reported wild-type S protein selective aptamer C9 was truncated (C9t) by its primer sequence [25] and its functionality as a bioreceptor on flexMEA chips for an electrochemical biosensing application was analyzed.

#### 3.1.1. Density Calculation

As a first step, the density of the immobilized aptamers on the surface of the flexMEA chips was evaluated by chronocoulometry (CC) and calculated to be $\Gamma_{DNA}=\left(1.3\pm 0.3\right)\times 10^{12}\;molecules/cm^{2}\;$, showing values in accordance with previously reported functionalized gold electrodes [44]. The initial consideration was that with the utilization of a new modified di-DTPA anchoring molecule, a reduced aptamer density might occur, as these groups occupy more of the available binding sites compared to single thiol aptamers. However, no noteworthy diminishing of the aptamer density was measured, suggesting the suitability of di-DTPA as an anchoring unit for aptasensor applications due to the high stability of the fourfold gold–thiol binding.

#### 3.1.2. QCM-D Investigation

**Figure 1:**
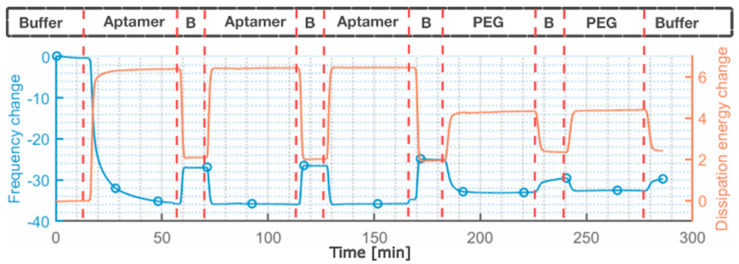
QCM-D measurements show the change of frequency in blue, and dissipation energy in orange. The aptamer solution flowed into the chamber in a 30 min window and was then cleaned with a buffer for 10 min. This procedure was repeated three times. For the PEG solution, two of these procedures were conducted. No change after the first buffer cleaning was observed for either. B stands for Buffer.

To determine the immobilization of the molecules and the optimal immobilization time for the functionalization of the flexMEA electrodes (Figure 1) with the multi-thiol aptamer, the common incubation method was tested by monitoring the real-time change in the mass on the surface of the electrode by QCM-D. Firstly, the functionalization step with the C9t aptamer led to a mass change of $\Delta m_{C9t}=7.79\pm 0.28\;ng/mm^{2}$. After the functionalization with PEG, the mass increased further by $\Delta m_{PEG}=1.42\pm 0.05\;ng/mm^{2}$. Of the total mass added to the electrode, the PEG addition amounts to 15%, pointing to a surface covered mostly with aptamers instead of backfill molecules.

Under the investigated conditions, one 30 min exposure to either functionalization molecule was already sufficient to achieve a steady state on the surface, where the resonance frequency Δf stayed constant. The short incubation time required to establish a steady state adlayer can be explained by the immobilization via di-DTPA, which possesses four thiols and thus a higher binding probability for the aptamer to the Au electrode. For the further characterization of the aptasensors, a longer exposure time of two hours was chosen [45,46,47]. This was carried out to ensure the comparability of the fabricated aptasensors with the literature. Further confirmation of the biofunctionalization was demonstrated by XPS analysis (Table S1, Figure S2).

#### 3.1.3. SPR Investigation

**Figure 2:**
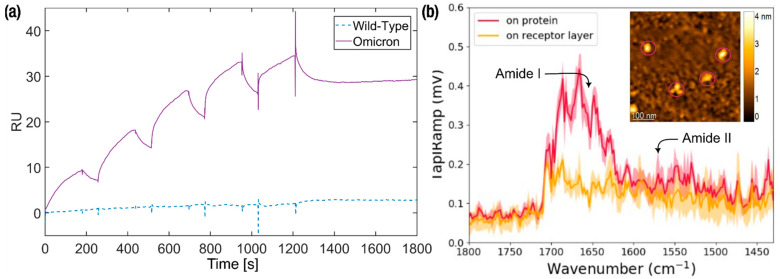
SPR and AFM-IR investigations. (**a**) SPR measurements with concentration series of 55, 166, 500, 1500, and 3000 nM of the truncated C9t aptamer for binding affinity determination to wild-type (dash line) and Omicron (continuous line) S proteins. (**b**) AFM-IR topography (inset) and spectra of aptamer C9t/PEG layer and S protein. The inset shows the AFM height image with the S protein–C9t aptamer complexes (circled) resolved. Spectra recorded of the S protein–C9t complex (circled) exhibit a strong feature assigned to the amide I band, followed by a broad feature at lower wave numbers assigned to the amide II band of protein. The spectra are averaged from the four circled positions and four positions on the receptor layer.

The ability of the 40-base truncated aptamer to bind the Omicron S protein was corroborated by SPR experiments, while surprisingly only a weak association was observed for its originally reported target wild-type S protein (Figure 2a). The kinetic parameters for the interaction between C9t and Omicron S protein were derived (Table 1). The association rate constant (*k*_a_) exhibited a mean value of $1.48\cdot 10^{4}\pm 1.33\cdot 10^{3}$ (1/Ms). Similarly, the dissociation rate constant (*k*_d_) had a mean value of $2.21\cdot 10^{-4}\pm 9.23\cdot 10^{-5}$(1/s). The resulting equilibrium dissociation constant (*K*_D_) was 14.6±4.7 nM, being well within the range of similar aptamers for the Omicron S protein [48] as well as other aptamers for S proteins of different SARS-CoV-2 variants [49]. Furthermore, the here-found *K*_D_ is well below the previously found *K*_D_ of 230 nM for the full-length C9 aptamer targeting the wild-type variant, hinting at a higher affinity between the truncated version C9t in contrast to its full-length version C9 [25].

#### 3.1.4. AFM-IR Investigation

To confirm the binding of the C9t aptamer/PEG receptor layer to the Omicron S protein of the SARS-CoV-2 virus, AFM-IR investigations were performed. This technique provides the capability of characterizing the composition of the sample by both topographic and spectroscopic properties on the level of individual molecules [43]. The binding of protein to the receptor layer was resolved by tapping AFM-IR (Figure 2b). The inset of Figure 2b represents the topography image recorded after protein incubation. Globular features with an average height of approx. 4 nm were observed. To identify the chemical composition of features on the surface, nano-IR point spectra were recorded on the four objects (pink circles) and four points next to them on the functionalized gold surface. In the spectra recorded of the high features, slightly increased absorbance appears at 1520–1560 cm^−1^ together with a significantly stronger band between 1607–1710 cm^−1^. Together these bands can be assigned to amide II and amide I bands of the attached protein [50]. The amide bands are absent in the spectra recorded of the surface next to the topographic features, which was presumably the receptor layer. Therefore, the circled features are proven complexes of the S protein and the C9t aptamer (Figure 2b). These preliminary findings are further substantiated in a detailed investigation by AFM-IR experiments to elucidate the structural and functional relation of the receptor layer and the S protein [51].

### 3.2. Aptasensor Fabrication and Performance Results

The use of the flexMEA allows individual sensor signals, thus, redundant measurements, that corroborate the detection and discard the possibility of a false-positive result as demonstrated previously in two other disease applications [27,52]. To evaluate the aptasensor performance on the flexMEA chips, its calibration curves were determined in different media. The binding of the target to the aptamer receptor layer induced a conformational change in the aptamer, thus modifying the ferri/ferrocyanide charge transfer characteristics, which were registered by differential pulse voltammetry (DPV). DPV was employed as a detection technique since this method applies superimposed small-amplitude potential pulses, which decreases the effect of the charging current providing a sharper peak signal corresponding to the redox probes. Here, an increase in the peak current signal was observed as the concentration of the protein rose. The current increase can be understood as a result of a reduced charge transfer resistance as previously explained due to conformational rearrangements within the receptor layer.

#### 3.2.1. Calibration Curves

**Figure 3:**
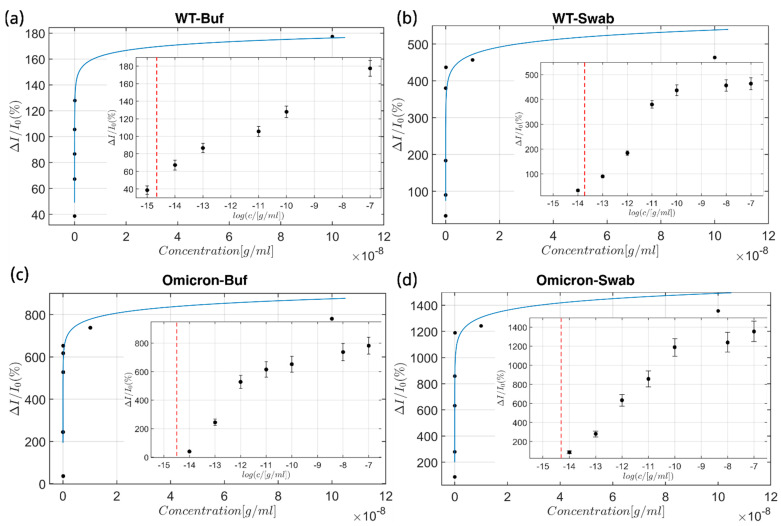
Calibration curves. The calibration curves represent the response of the aptasensor in a concentration ranging from 1 fg/mL to 100 ng/mL of the target proteins: against (**a**) wild-type S protein of the SARS-CoV-2 virus in ferri/ferrocyanide solution and (**b**) wild-type S protein of the SARS-CoV-2 in a spiked negative control (non-infectious) nasal swab, (**c**) the S protein of the Omicron variant in ferri/ferrocyanide solution, and (**d**) the S protein of the Omicron variant in a spiked negative (non-infectious) control nasal swab. The overlay figure shows the Langmuir–Freundlich curve representation of the biosensor’s response without logarithmic axis modifications, whilst the inset shows the logarithmic representation of the obtained data, and the dashed line represents the LoD for the individual media–analyte combination. Hereby, all calibration curves were determined at 16 individual electrodes per chip, for three chips per condition.

The response of the aptasensor was determined by DPV measurements against S protein of wild-type and Omicron variants of SARS-CoV-2 virus in tris-HCl buffer solution and spiked negative nasopharyngeal swab (non-infectious) samples, all containing ferri/ferrocyanide as redox tracers (Figure 3).

The calibration curves were determined for concentrations ranging from 1 fg/mL to 100 ng/mL of the target proteins. As a reference, the calibration curve for the binding of the wild-type S protein by the C9t aptasensor in ferri/ferrocyanide solution can be described by the equation $\Delta I/I_{0}\;(\%)=17.1\cdot log(c_{i})+298.8$. The calculated limit of detection (LoD) of 3.8 fg/mL (Figure 3a) was obtained as three times the standard deviation of the normalized electrochemical signal from the blank. As the blank is used as a reference the signal value of the lowest measured concentration and its deviation is used for the LoD calculation [53]. The binding of the wild-type S protein in the nasopharyngeal fluid control sample can be represented by the following equation: $\Delta I/I_{0}\;(\%)=109.7\cdot log(c_{i})+1540.7$ and an LoD of 21.6 fg/mL (Figure 3b). For the Omicron S protein in buffer, the following equation can be used: $\Delta I/I_{0}\;(\%)=160.35\cdot log(c_{i})+2339.4$ with a LoD of 4.9 fg/mL (Figure 3c). Lastly, the calibration curve of the Omicron S protein in the control nasal swab was best delineated by the following equation: $\Delta I/I_{0}\;(\%)=209.5\cdot log(c_{i})+3089.0$ with a LoD of 8.0 fg/mL (Figure 3d).

#### 3.2.2. Selectivity Analysis

**Figure 4:**
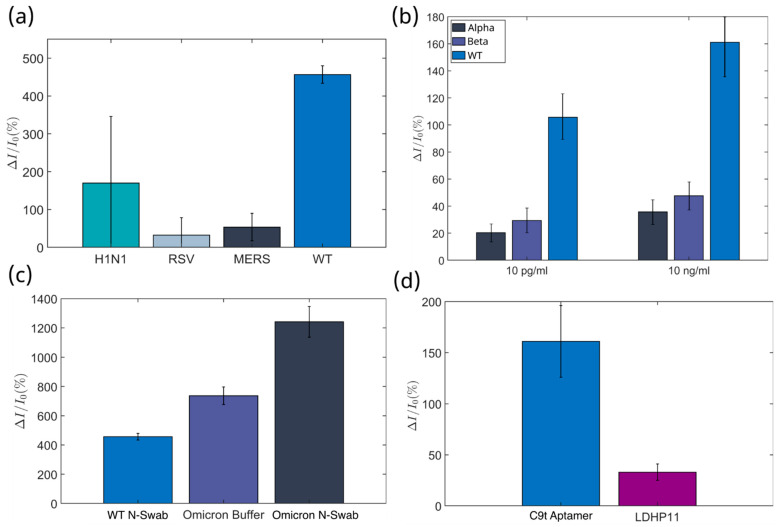
Selectivity Tests for the Aptasensor. The aptasensor’s response to analog analytes (selectivity tests) was conducted at an analyte concentration of 10 ng/mL. The selectivity test was performed as the responses to (**a**) wild-type response for viral proteins of other respiratory viruses with similar symptoms, (**b**) different strains of the SARS-CoV-2 virus, and (**c**) between wild-type and the Omicron variant, in ferri/ferrocyanide solution (Buffer), as well as in spiked negative nasal swab samples (N-Swab). (**d**) Comparison of the detection response in buffer for Omicron S protein by the C9t aptasensor versus a previously reported malaria aptamer LDHp11 [54]. Hereby, all selectivity tests were conducted at 16 individual electrodes per chip, for two chips per condition.

Furthermore, the C9t aptasensor showed a high selectivity for S proteins of the wild-type and Omicron variants in comparison to closely related off-targets like coronavirus MERS-CoV and biomarkers of respiratory viruses with similar symptoms to COVID-19, namely RSV and Influenza A H1N1 (Figure 4a). With a high analyte concentration of 10 ng/mL, the response against a target protein of Influenza A H1N1 was $\Delta I/I_{0}=170.0\pm 176.0$, a target protein of RSV was $\Delta I/I_{0}=32.1\pm 46.5$, and against the S protein of the MERS-CoV virus was $\Delta I/I_{0}=53.4\pm 36.4,$ whereas the response to the Omicron SARS-CoV-2 S protein was $\Delta I/I_{n}\approx 1241\pm 105.1$. This represents a 42, 24, and 8 times higher response to the Omicron analyte than to the other analog proteins. This is also valid for the wild-type S protein. Although the response to the latter was lower than that of the Omicron S protein, it represents a 14, 8, and 2.7 times higher response versus those analog proteins. All tested alternative analytes showed a response below the signal of the LoD at $\Delta I/I_{0}=177.3$ for SARS-CoV-2 wild-type and Omicron in the nasopharyngeal swab solution. This ensures the capability of the C9t aptasensor to differentiate between the SARS-CoV2 S Omicron protein variant and other respiratory viruses, which poses a diagnostic challenge due to their similar symptoms.

The selectivity of an aptasensor was further evaluated against variants of the S protein of the SARS-CoV-2 virus. Using a concentration of 10 ng/mL, a response for the S protein of the wild-type variant resulted in $\Delta I/I_{0}=161.0\pm 35.3$ in the buffer solution, and $\Delta I/I_{0}=456.7\pm 23.2\;$in the negative nasopharyngeal swab. The highest response was measured against the S protein of the Omicron variant with $\Delta I/I_{0}=736.6\pm 60.2$ in buffer solution and $\Delta I/I_{0}=1241.2\pm 105$ in the negative nasopharyngeal swab (Figure 4c). The widespread alpha and beta variants were evaluated with their S proteins in a buffer solution, and a low response was found compared to the response against the S protein wild-type variant. In the case of the S protein of the alpha variant, the response was$\;\Delta I/I_{0}=17.89\pm 5.15$ and $\Delta I/I_{0}=31.90\pm 8.61$ with 85% and 78% smaller signals for 10 pg/mL and 10 ng/mL, respectively. Furthermore, for the S protein of the beta variant, the responses $(\Delta I/I_{0}=27.15\pm 8.36\;$and $\Delta I/I_{0}=43.65\pm 12.75)$ were 79% and 70% smaller (Figure 4b). Additionally, control experiments demonstrated that a specific aptamer sequence of C9t obtained 4.8 times higher signals for Wildtype S protein detection and 24.5 times higher signals for Omicron S protein detection than the LDHp11 aptamer used as control and previously reported for the detection of a malaria biomarker (Figure 4d) [27].

## 4. Discussion

### 4.1. Aptasensor Physicochemical Characterization

The truncation of the previously reported C9 aptamer was intended for its implementation as a receptor molecule in an electrochemical transducer to facilitate receptor-analyte detection on the surface of the electrode. Resilience in analyte binding to truncation is occasionally reported in the literature, however, aptamer–protein interactions are complex and difficult to predict. The truncated C9t aptamer applied here sustained its robust binding affinity even after the elimination of 40 nucleotides from its primer regions. The aptamer surface density on the electrode remained unaltered in comparison with single-thiol aptamer upon the modification with the novel di-DTPA (four-thiol) terminal molecule, suggesting the suitability of such a molecule as a stable anchoring unit. The immobilization of all the molecules and S protein detection were demonstrated by QCM-D, XPS, and AFM-IR analysis. This latter corroborated also the C9t aptamer/S protein interaction at the molecular level by the determined amide bonds of the protein. Moreover, even if originally intended for use with the wild-type variant [25], the C9t aptamer showed a higher affinity towards the Omicron variant corroborated by its almost 16 times higher *K*_D_ determined by SPR. Therefore, these preliminary findings warrant further, more detailed characterization to fully elucidate the structural and functional underpinnings of these observed binding behaviors.

### 4.2. Aptasensor Fabrication and Performance Evaluation

The lowest clinically relevant concentration of the S protein of the SARS-CoV-2 virus amounts to 10.3 fg/mL [55,56]. This confirms that the obtained LoDs for the aptasensor C9t remain below the clinically relevant S protein concentrations in all media, except for the wild-type in a spiked negative control nasal swab sample, whereby the order of magnitude is still in the same range (LoD of 21.6 fg/mL). Our observations highlight the importance of the selection medium on the output result of the aptasensors since the aptamer binding capability is dictated by the selection medium. Furthermore, the aptasensor showed a high sensitivity of 17.1, 109.7, 160.3, and 209.5 per concentration decade for the different test settings shown in Figure 3a,b,c,d, respectively. These sensitivities are higher than previously reported electrochemical biosensors, which range from 7.8 [19] and 8.1 [17] to 14.9 [16] per concentration decade (Table 2). The dynamic range for the four sets of experiments was more extensive in comparison to other biosensors for the same target analyte, covering 3.8 fg/mL–100 ng/mL, 21.6 fg/mL–7.4 ng/mL, 4.9 fg/mL–4.03 ng/mL, and 8.0 fg/mL–38 ng/mL, as shown in Figure 3a,b,c,d, respectively.

In comparison to previously reported biosensors, our aptasensor would suffice to quantify a viral load by matching best with the clinically relevant range by covering from 0.3 fg/mL to 13.7 ng/mL [55,56]. Furthermore, in comparison to commercial rapid test kits, this aptasensor surpasses the general LoD of those tests of 40 ng/mL and should fulfill the PCR standard at 30 pg/mL [55,56,57]. Noteworthy, this sensor is more sensitive to Omicron than wild-type S protein but still outperforms other aptasensors optimized for wild-type S protein detection (See Table 2).

The different affinities of the C9t aptamer for various SARS-CoV-2 variants hint at the influence of the viral evolution on the binding capabilities of the aptamer. The aptamer’s inability to recognize the Alpha and Beta variants, contrasted with its enhanced affinity for the Omicron variant, suggests that the mutations in Alpha and Beta may have compromised the accessibility of the aptamer’s binding domain. In contrast, the mutations in the Omicron variant seem to have augmented this accessibility for the C9t aptamer. This varied response to different variants of the same virus (a strong response to Omicron and lower to both Alpha and Beta in comparison to the wild-type) underlines the high variability of aptasensors and the importance of characterizing them using different viruses and variants, as their selectivity may vary depending on the variants and its changes. This is also seen in the work of other groups, e.g., recently reported aptamers showed changes in their binding affinity with changing *K*_D_ against different variants of the same virus, in part dependent on the different targets, which were used during the selection process [49]. This diverse sensitivity towards different variants of the same virus could be used to our advantage in a kind of logic gate configuration and as a variant-selective aptasensor [27].

During a pandemic, new viral variants can emerge rapidly, underscoring the need for diagnostic methods that can be adapted quickly. The conventional SELEX process for aptamer selection may take 2–8 weeks but advances in high-throughput, microfluidic, capillary electrophoresis-based, and automated platforms can shorten this to as little as 1–3 weeks [58]. Once an aptamer pool is generated, it can be optimized to bind emerging variants through additional rounds of SELEX, avoiding the need to start the entire selection from scratch [59]. Because aptamers are chemically synthesized, they can be produced rapidly and at scale, making them suitable for point-of-care diagnostics when swift detection of new variants is crucial. While aptamers alone cannot fully prevent an outbreak, their adaptability, cost-effectiveness, and fast development process significantly strengthen the toolbox for controlling rapidly evolving viral threats.

## 5. Conclusions

Through SPR and AFM-IR, it was demonstrated that even if the primers were truncated from the aptamer, their binding capability was conserved, indicating that these primers had only negligible influence on the functionality of the C9 aptamer. The immobilization procedure of the aptamer with a di-DTPA binding group resulted in a short adsorption time of 30 min, with a similar aptamer density to single-thiol ssDNA, promising higher stability and sparseness, whilst not reducing its binding capabilities.

Furthermore, the truncated multi-thiol aptasensor detected the target S protein of the wild-type and Omicron variants of SARS-CoV-2 with a low LoD in spiked ferri/ferrocyanide buffer solution and a nasal swab sample, at clinically relevant concentrations. This confirms its usability as a single-use PoC aptasensor. Additionally, the C9t aptasensor showed a wide dynamic range from fg/mL to ng/mL. The high selectivity for Omicron S protein over other protein-based biomarkers for infectious diseases and even over other S proteins from other SARS-CoV-2 variants enables a variant selective detection of infections with Omicron SARS-CoV-2 viruses. Future work will be towards the characterization of the aptasensor with VLPs (virus-like particles) and collecting samples from patients. Additionally, stability testing and comparing the effect of single and di-dithiol functionalization are part of this future work.

The low costs from the truncated aptamer and flexMEA chip and the fast sensor assembly paired with high sensitivity and variant-specific selectivity render the reported aptasensor an affordable and reliable testing tool to manage future fast-spreading infections with its quantification capabilities that outperform current rapid diagnosis tests.

## Figures and Tables

**Table 1:** SPR-determined characteristic values of the aptamer C9t for the Omicron variant of the S protein.

Metric	Value (Mean + SD)
**ka (1/Ms)**	$(1.48\pm 0.1)\times 10^{4}$
**kd (1/s)**	$(2.21\pm 0.9)\times 10^{-4}$
**K_D_ (M)**	$(1.46\pm 0.5)\times 10^{-8}$

**Table 2:** Comparison between characteristic values of different electrochemical aptasensors.

This Work	Limit of Detection	Sensitivity per Decade	Dynamic Range
(A) WB	3.8 fg/mL	17.1 ± 0.8	3.8 fg/mL–100.0 ng/mL
(B) WC	21.6 fg/mL	109.7 ± 13.2	21.6 fg/mL–7.4 ng/mL
(C) OB	4.9 fg/mL	160.3 ± 29.0	4.9 fg/mL–4.0 ng/mL
(D) OC	8.0 fg/mL	209.5 ± 30.7	8.0 fg/mL–38.0 ng/mL
Other works/references			
[16]	66.0 pg/mL	14.9	66.0 pg/mL–1.33 µg/mL
[17]	0.8 pg/mL	7.8	0.8 pg/mL–1.0 µg/mL
[18]	19.0 ng/mL	-	19.0 ng/mL–2.0 µg/mL
[7]	44-0 ag/mL	-	44.0 ag/mL–100.0 pg/mL
[19]	116 fg/mL	8.1	1.0 pg/mL–10.0 ng/mL

W = wild-type; O = Omicron; B = buffer; C = nasopharyngeal swab.

## Data Availability

The raw data supporting the conclusions of this article will be made available by the authors upon request.

## Supplementary Materials

The following supporting information can be downloaded at: https://www.mdpi.com/article/10.3390/bios15010024/s1, Figure S1: Schematic of the flexible polymer-based S protein aptasensor array (flexMEA sensor chip); Table S1: XPS proportion results; Figure S2: XPS analysis of bare gold electrode and functionalized aptasensor.
