# Supplementary material for: A Truncated Multi-Thiol Aptamer-Based SARS-CoV-2 Electrochemical Biosensor: Towards Variant-Specific Point-of-Care Detection with Optimized Fabrication
Source: Biosensors (Basel). 2025 Jan 6;15(1):24. doi: 10.3390/bios15010024 (PMC11763500; doi:10.3390/bios15010024)
Supplement: Supplementary file 1 [file biosensors-15-00024-s001.zip › biosensors-3392694-supplementary.pdf]

Supplemental Material

A truncated multi-thiol aptamer-based SARS-CoV-2 electrochemical biosensor: Towards Variant-Specific Point-of-Care Detection with Optimized Fabrication.

Sergio Roberto Molina Ramirez [1] , Nafiseh Samiseresht [2] , Mateo Alejandro Martínez-Roque [1] , Ferdinando Catania [1] , Kevin Graef [1] , Martin Rabe [2] , Andreas Offenhäusser [1] , Dirk Mayer [1] and Gabriela Figueroa-Miranda1 ,*

1 Institute of Biological Information Processing, Bioelectronics (IBI-3), Forschungszentrum Jülich GmbH, 52428 Jülich, Germany
2 Department of Interface Chemistry and Surface Engineering, Max Planck Institute for Sustainable Materials GmbH, 40237 Düsseldorf, Germany
* Correspondence: g.figueroa.miranda@fz-juelich.de

S1. Materials and Methods

S1.1. Flexible multi-electrode Array (flexMEA) Fabrication

Briefly, on a substrate of polyethylene terephthalate (PET, DuPont Teijin Films Ltd), a 5 nm titanium adhesion layer and a 50 nm gold layer were deposited through physical vapor deposition (Pfeiffer PLS 570, Pfeiffer Vacuum). The feedlines and patterned electrodes were fabricated via standard photolithography using a Mask aligner (MA4, Süss MicroTec). The excess materials were removed through chemical etching. Lastly, to remove the photoresist layer, the flexMEAs were immersed in acetone and isopropanol for 10 minutes each. The feedlines were then passivated by photolithography with Parylene-C. [1]

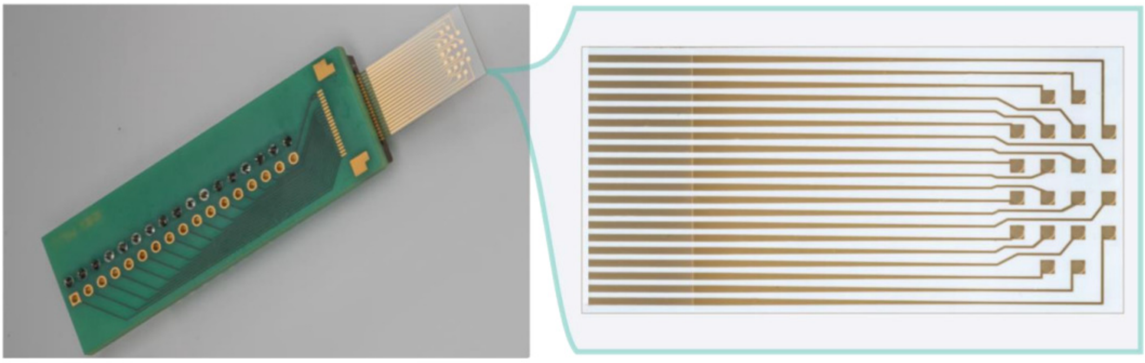

Figure S1. Schematic of the flexible polymer-based S protein aptasensor array (flexMEA sensor chip). Fabricated flexible multi-electrode array sensor chip on ZIF connector (left) and full image of the electrode array showing the individually addressable electrodes (right).

S1.2. X-Ray Photoelectron Spectroscopy (XPS)

Two different samples were prepared using square electrodes of 1 cm x 1 cm, with a 10 nm layer of titanium and a 50 nm layer of gold. All samples were subjected to the same chemical and electrochemical

cleaning as previously described. The analysis was conducted using a Phi5000 VersaProbe II spectrometer (ULVAC-Phi Inc., Kanagawa, Japan) with an Al K-alpha, monochromatic source (1.486 keV). The X-ray settings were 50 W, 15 kV, and a spot of 200 µm. The survey spectra were recorded with a 187.5 eV pass energy, a step of 0.8 eV, and a rate of 100 ms/step.

S2. Results

S.2.1 XPS Investigation

XPS was used to confirm the chemical composition of the formed self-assembled monolayer (SAM) on the surface of the gold electrode, resulting in a ratio of the present elements within the uppermost surface layer (approx. 10 nm) of the electrode (Figure S2). For each differently modified electrode, three discrete positions were measured. In each electrode, the surveys targeted a total of five different elements namely gold (Au 4f), oxygen (O 1s), nitrogen (N 1s), phosphorus (P 2p), and carbon (C 1s) (Table S1). First, a bare gold electrode without any functionalization was measured for reference and three elements were detected: Au 4f, C 1s, and O 1s. The latter two can be assigned to traces of contamination on the sample or on the electron optics.

The functionalized electrodes with both the aptamer C9t and PEG resulted in a lower Au 4f proportion due to the additional layer formation and a corresponding attenuation of the Au 4f photoelectrons. Furthermore, the appearance of N 1s and P 2p peaks confirmed the aptamer immobilization as they are the main elements of ssDNA. The increment of C 1s and O 1s can be assigned to the immobilization of both molecules.

Table S1. XPS proportion results.

METRIC	AU 4F	C 1S	O 1S	N 1S	P 2P
BARE	54.7 ± 0.8	36.3 ± 0.8	8.9 ± 0.1		
FUNC	14.9 ± 0.6	47.8 ± 0.3	26.9 ± 0.1	5.4 ± 0.3	2.0 ± 0.1

BARE: Bare Electrode, FUNC: Functionalized Aptasensors.

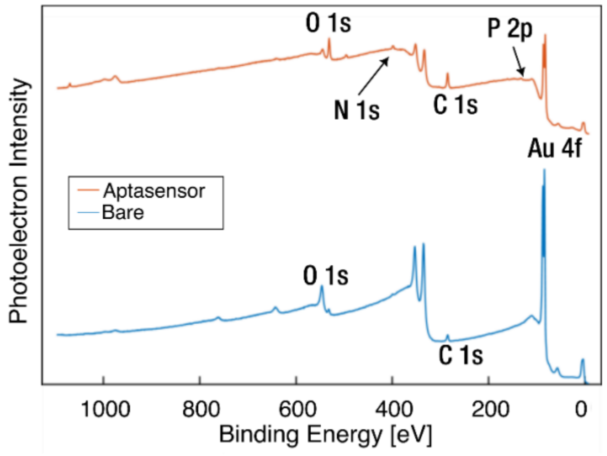

Figure S2. XPS analysis of bare gold electrode and functionalized aptasensor. In each graph, the peaks of the found elements are added. Gold (Au 4f, 84 eV), carbon (C 1s, 285 eV), oxygen (O 1s, 533 eV), nitrogen (N 1s, 400 eV), and phosphorous (P 2p, 134 eV) [2]. Furthermore, the y-values are shifted for clarity (bare by 1000, aptasensor by 3000).

References

1. Figueroa-Miranda, G.; Chen, S.; Neis, M.; Zhou, L.; Zhang, Y.; Lo, Y.; Tanner, J.A.; Kreidenweiss, A.; Offenhäusser, A.; Mayer, D. Multi-target electrochemical malaria aptasensor on flexible multielectrode arrays for detection in malaria parasite blood samples. Sensors and Actuators B: Chemical 2021, 349, 130812, doi:10.1016/j.snb.2021.130812.

2. Figueroa-Miranda, G.; Wu, C.; Zhang, Y.; Nörbel, L.; Lo, Y.; Tanner, J.A.; Elling, L.; Offenhäusser, A.; Mayer, D. Polyethylene glycol-mediated blocking and monolayer morphology of an electrochemical aptasensor for malaria biomarker detection in human serum. Bioelectrochemistry 2020, 136, 107589, doi:10.1016/j.bioelechem.2020.107589.

Disclaimer/Publisher's Note: The statements, opinions, and data contained in all publications are solely those of the individual author(s) and contributor(s) and not of MDPI and/or the editor(s). MDPI and/or the editor(s) disclaim responsibility for any injury to people or property resulting from any ideas, methods, instructions, or products referred to in the content.
